# Methicillin-Resistant *Staphylococcus aureus* in a Beauty Salon, the Netherlands

**DOI:** 10.3201/eid1411.071297

**Published:** 2008-11

**Authors:** Xander W. Huijsdens, Maria Janssen, Nicole H.M. Renders, Alexander Leenders, Paul van Wijk, Marga G. van Santen-Verheuvel, Jolanda Koel van-Driel, Gabriella Morroy

**Affiliations:** National Institute for Public Health and the Environment, Bilthoven, the Netherlands (X.W. Huijsdens, M.G. van Santen-Verheuvel); Jeroen Bosch Hospital, ‘s-Hertogenbosch, the Netherlands (M. Janssen, N.H.M. Renders, A. Leenders, P. van Wijk); De Gemeenschappelijke Gezondheidsdienst Hart voor Brabant, ‘s-Hertogenbosch (P. van Wijk, J.K. van Driel, G. Morroy)

**Keywords:** methicillin resistance, Staphylococcus aureus, community-associated infections, Panton-Valentine leukocidin, dispatch

## Abstract

An outbreak of community-associated USA300 methicillin-resistant *Staphylococcus aureus* occurred in a beautician and 2 of her customers. Eight other persons, who were either infected (n = 5) or colonized (n = 3), were linked to this outbreak, including a family member, a household contact, and partners of customers.

The reported number of community-associated methicillin-resistant *Staphylococcus aureus* (CA-MRSA) infections is increasing rapidly. CA-MRSA is increasingly isolated from patients who lack traditional risk factors for colonization or infection. CA-MRSA often contains the virulence factor Panton-Valentine leukocidin (PVL), which causes skin and soft tissue infections.

The CA-MRSA USA300 strain is known to cause outbreaks among population groups (*1*), such as native Americans, prison inmates, military personnel, men who have sex with men, and competitive sports participants, and accounts for 97% of MRSA isolates obtained in emergency departments across the United States from patients with soft tissue infections (*2*). CA-MRSA is associated with invasive infections, including necrotizing fasciitis (*3*), sepsis (*4*), and pneumonia (*5*). The USA300 strain, which is also found in Europe (*6*), was first isolated in the Netherlands in 2002.

Overall prevalence of MRSA in the Netherlands is low (2%) (*7*). In 2006, 3.8% (n = 76) of all MRSA isolates (1 per patient) sent to the National Institute for Public Health were identified as the USA300 strain. We report an outbreak of the USA300 strain related to a beauty salon in the Netherlands, in a beautician, a family member, a household contact, and customers and their partners.

## The Study

In September 2005, a medical microbiologist from the regional medical microbiology laboratory reported to the municipal health department a recurring MRSA infection in a beautician. From December 2004 onwards, the woman had recurrent infections on the legs, buttocks, and groin resulting in incision and drainage of lesions. When an abscess developed in the genital area in July 2005, MRSA was cultured from a wound swab. In December 2005, the beautician was declared MRSA-free after antimicrobial treatment. Swabs were taken 3 times in 1-week intervals from nose, throat, perineum, and wound and used for enrichment culture of MRSA. In March 2006, the woman was tested again for MRSA colonization; test results showed that she had been reinfected or that therapy had failed. The beautician also had eczema. Because of the “hands on” nature of her work, she was advised to temporarily stop providing services to customers.

The municipal health department conducted a risk assessment of the woman’s household contacts and the beauty salon. The Netherlands does not require that MRSA infections be reported. Therefore, the municipal health department depends upon the consent and full cooperation of index patients and contacts for further investigation of outbreaks. Consequently, in this instance, household contacts for screening were identified but had not presented themselves for screening. Contacts who had complaints sought treatment at the emergency department, where the observant infection control practitioner (ICP) and microbiologists related them to the MRSA outbreak. Nurses obtained specimens by swabbing each patient’s nose, throat, and wounds. A case was defined as a patient who had a culture-confirmed MRSA infection during the outbreak period July 2005–December 2006 and a direct epidemiologic link to the index patient.

In April 2006, a salon customer was hospitalized with an abscess of the breast caused by MRSA; in July 2006, another customer who had had boils since February 2006 was found to be MRSA positive. Both customers had been given wax treatments by the beautician during the period in which she had an infected hair follicle in her armpit. Swabs taken from this site showed that the beautician was infected with the same MRSA strain as before. Concern arose about the risk for infection to customers through instruments, materials (wax), or contact with other employees. The index patient and the other 6 employees of the salon regularly provided services to each another.

A nurse and ICP of the municipal health department visited the salon in June 2006 to check on hygiene protocols and to advise on preventive measures to reduce risk for further transmission. All working procedures and protocols were investigated, and the salon was advised to clean and disinfect instruments and procedure rooms. More specifically, the ICP observed a total waxing procedure performed by the staff. Ten swabs were taken from used wax, wax implements, and the treatment room. All 6 employees were screened and informed about MRSA and the current situation. Arrangements were also made to test 22 regular customers who had received wax treatments by the index patient in the previous 2 months. In the following weeks, these customers were screened at the municipal health office and informed about MRSA. Of the 22 regular customers, 21 completed a questionnaire and 19 were actually screened for MRSA by culturing samples from nares and throats.

All employees and the 19 selected regular customers were negative for MRSA colonization. All environmental swabs were also negative for MRSA. It was noted that the 70% alcohol used to disinfect the skin after waxing was diluted with water because customers had complained about the stinging effect of the alcohol on treated skin. Furthermore, it became apparent that after performing waxing treatments the beautician would touch the waxed skin of customers with ungloved hands to check for remaining hairs. She did not wash her hands after removing the gloves.

During the outbreak investigation, more background information became available from those who were MRSA colonized or infected and who could be indirectly linked to the beautician or her customers. During the week that the first infected customer was identified (in April 2006), another customer was hospitalized with an abscess in the groin. Unfortunately, no culture was taken from this patient. The partner of the second infected customer was also infected with MRSA that was related to an abscess on his leg. By the end of 2006, a MRSA-positive couple was identified as a contact of the second infected customer. In August 2006 another couple was reported to be MRSA positive; both had abscesses on the thighs. Because no further epidemiologic data could be obtained, whether the couple’s infection was linked to the beauty salon is not clear.

A total of 45 persons who had been in direct or indirect contact with the beautician were screened for MRSA: 3 family members, 3 roommates, 11 other persons (including secondary contacts), 6 beauty salon employees, and 22 customers (including regular customers). Fifteen persons had skin infections and 10 of them were colonized with MRSA (beautician, family member, roommate, ex-partner of the roommate, customers, and partners of customers). Although skin infections never developed in the beautician’s family members, tests did show MRSA colonization in one of them. The beautician’s boyfriend, a native of the United States, had already lived for >2 years in the Netherlands. Although he had skin lesions, no *S. aureus* was found. The girlfriend of a sport mate who regularly exercised with the partner of a customer was colonized with MRSA at the end of 2006. She had immigrated recently from the United States to the Netherlands, but her first screening test results were negative. The mean age of the patients was 29 years (range 21–40 years).

Eleven people were found to be MRSA positive. Of these 11, 3 persons with a direct link to the beauty salon (the beautician and 2 customers) (Table), 6 with an indirect link (family member, roommate, ex-partner of roommate, partner of a customer, sport mate of partner of a customer and his partner), and a couple from whom no epidemiologic data could be obtained were infected with the same MRSA strain as the beautician. To characterize the MRSA isolates, the following typing methods were used: pulsed-field gel electrophoresis (PFGE), staphylococcal protein A (*spa*) typing, multilocus sequence typing, staphylococcal cassette chromosome *mec* (SCC*mec*) typing, PCR for PVL genes (*LukS*-*LukF*), and a recently described assay for the USA300 strain-specific arginine catabolic mobile element (ACME)–encoded *arcA* gene (*8*).

**Table:** Characteristics of MRSA outbreak related to a beauty salon, the Netherlands*

Case no.	Case-patient	Age, y	Gender	Type of infection	Site of infection	Date of first positive MRSA culture	MRSA sample test results
1	Beautician, index	21	F	Abscess	Leg, buttock, groin	2005 Jul	W+
2	Customer no. 1†	36	F	Abscess	Breast	2006 Mar	W+
3	Customer no. 2	29	F	Boils, abscess	Genitals	2006 Jul	N+, T+, P+, W+

*MRSA, methicillin-resistant *Staphylococcus aureus*; W, wound; N, nose; T, throat; P, perineum; +, positive. †Hospitalized.

All MRSA isolates were identical and identified as the well-known CA-MRSA USA300 strain. All strains were PFGE type 218 (according to the Dutch PFGE classification system = USA300), *spa* type t024, sequence type (ST)8 (1 isolate was characterized as ST879, a singleton of ST8), SCC*mec* IVa, and positive for PVL and ACME. All MRSA isolates had identical susceptibility patterns: resistant to oxacillin (and thus to all β-lactam antimicrobial drugs) and erythromycin, and susceptible to rifampicin, ciprofloxacin, gentamicin, clindamycin, vancomycin, teicoplanin, tetracycline, cotrimoxazole, mupirocin, and fusidic acid.

## Conclusions

Outbreaks of CA-MRSA strains have been reported with increased frequency. Several reports involved outbreaks among competitive sports participants, military personnel, men who have sex with men, prisoners, native Americans, and drug users (*1*,*9*,*10*). Skin treatments in a beauty salon likely led to MRSA transmission as a result of contact with an infected beautician.

Clearly, our study and others show that CA-MRSA is an emerging problem in the community setting. In the Netherlands, patients are generally only tested after recurrent infections. Unless outbreaks occur in a defined group, MRSA remains undetected in the general population because reporting is not mandatory. Although the prevalence of MRSA in the Netherlands is low, local microbiologic laboratories should report outbreaks, when detected, to the local municipal health department for further investigation. More research is necessary to better understand the risk factors involved in these outbreaks.
